# Infections in Deep Brain Stimulator Surgery

**DOI:** 10.7759/cureus.5440

**Published:** 2019-08-20

**Authors:** Jacob E Bernstein, Samir Kashyap, Kevin Ray, Ajay Ananda

**Affiliations:** 1 Neurosurgery, Riverside University Health System Medical Center, Moreno Valley, USA; 2 Neurosurgery, Kaiser Permanente, Los Angeles, USA

**Keywords:** deep brain stimulator infection, dbs

## Abstract

Introduction: Deep brain stimulation has emerged as an effective treatment for movement disorders such as Parkinson’s disease, dystonia, and essential tremor with estimates of >100,000 deep brain stimulators (DBSs) implanted worldwide since 1980s. Infections rates vary widely in the literature with rates as high as 25%. Traditional management of infection after deep brain stimulation is systemic antibiotic therapy with wound incision and debridement (I&D) and removal of implanted DBS hardware. The aim of this study is to evaluate the infections occurring after DBS placement and implantable generator (IPG) placement in order to better prevent and manage these infections.

Materials/Methods: We conducted a retrospective review of 203 patients who underwent implantation of a DBS at a single institution. For initial electrode placement, patients underwent either unilateral or bilateral electrode placement with implantation of the IPG at the same surgery and IPG replacements occurred as necessary. For patients with unilateral electrodes, repeat surgery for placement of contralateral electrode was performed when desired. Preoperative preparation with ethyl alcohol occurred in all patients while use of intra-operative vancomycin powder was surgeon dependent. All patients received 24 hours of postoperative antibiotics. Primary endpoint was surgical wound infection or brain abscess located near the surgically implanted DBS leads. Infections were classified as early (<90 days) or late (>90 days). Infectious organisms were recorded based on intra-operative wound cultures. Number of lead implantations, IPG replacements and choice of presurgical, intra-operative, and postsurgical antibiotics were recorded and outcomes compared.

Results: Two hundred and three patients underwent 391 electrode insertions and 244 IPG replacements. Fourteen patients developed an infection (10 early versus 4 late); 12 after implantation surgery (3%) and 2 after IPG replacement surgery (0.8%). No intracranial abscesses were found. Most common sites were the chest and connector. *Staphylococcus aureus* (MSSA) was the most common organism. Intra-operative vancomycin powder did not decrease infection risk. Vancomycin powder use was shown to increase risk of infection after electrode implantation surgery (Relative Risk 5.5080, p = 0.02063). Complete hardware removal occurred in eight patients, one patient had electrode only removal, three patients with I&D and no removal of hardware, and two patients with removal of IPG and extensor cables only. All patients were treated with postoperative intravenous antibiotics and no recurrent infections were found in patients with hardware left in place.

Discussion/Conclusion: Infections after DBS implantation and IPG replacement occurred in 3% and 0.8% of patients respectively in our study which is lower than reported historically. Early infections were more common. No intracranial infections were found. Intra-operative use of vancomycin was not shown to decrease risk of infection after electrode implantation surgery or IPG replacement. However, in our study it was shown to increase risk of infection after electrode implantation surgery. Treatment includes antibiotic therapy and debridement with or without removal of hardware. DBS hardware can be safely left in place in select patients who may have significant adverse effects if it is removed.

## Introduction

Deep brain stimulation has emerged as an effective treatment for movement disorders such as Parkinson’s disease, dystonia, and essential tremor with estimates of >100,000 deep brain stimulators (DBSs) implanted worldwide since 1980s [1-2]. The applications of DBS continue to increase as it is now approved by the U.S. Food and Drug Administration (FDA) for epilepsy, and there are many reports of its efficacy in psychiatric disorders and pain syndromes [1]. Infection rates vary widely in the literature with rates as high as 25% [2-5]. Traditional management of DBS infections is systemic antibiotic therapy with wound incision and debridement (I&D) and removal of implanted DBS hardware [2, 4-5]. Removal of a DBS can be very devastating for patients and sometimes infections can be managed without removal of hardware [4-5]. Presurgical preparation with ethyl alcohol or use of intra-operative vancomycin showed to be effective in reducing infection rates in some studies, however, the results have been inconsistent [2, 6-7]. There are conflicting studies as to whether infection rates are higher after de-novo implantation surgery or battery replacement [8-9]. The aim of this study is to evaluate the infections occurring after DBS placement and implantable generator (IPG) placement in order to better prevent and manage these infections.

## Materials and methods

Methods

We conducted a retrospective review of 203 patients who underwent implantation of a DBS at a single institution between 2000 and 2018. During initial electrode placement, patients underwent either unilateral or bilateral electrode placement with placement of the IPG at the same surgery. IPG replacements occurred as a necessity when battery life was low or exhausted. For patients undergoing unilateral electrode placement, repeat surgery for contralateral electrode placement was performed after three months from the initial surgery if patients had bilateral symptoms. Cefazolin was the preoperative antibiotic of choice. Preoperative preparation with ethyl alcohol occurred in all patients while use of intra-operative vancomycin powder was surgeon dependent. Locations for vancomycin powder placement include the subgaleal space in the cranial incisions and in the subcutaneous pocket created for the IPG in the chest. All patients received 24 hours of postoperative antibiotics. Primary endpoint was surgical wound infection or brain abscess located adjacent to surgical site or along the tract of the implanted DBS leads. Infections were classified as early (<90 days) or late (>90 days). Infectious organisms were recorded based on intra-operative wound cultures. Number of lead implantations, IPG replacements, and choice of presurgical, intra-operative, and postsurgical antibiotics were recorded and outcomes compared. The difference in infection rates was compared and relative risk calculated with clinical significance indicated by a p value <0.05.

Infections were treated based on intra-operative cultures and with the guidance of an infectious disease specialist. Decisions to remove or leave hardware in place were made on a case by case basis and took into account the degree of infection seen during surgery, the clinical response, and the dependence of the patient on their DBS. Attempts to salvage the hardware were made when it would be a burden for the patient to not have their DBS or undergo subsequent surgeries.

## Results

In our study, 203 patients underwent 391 electrode insertions and 244 IPG replacements (Table 1). A total of 14 patients developed a hardware infection (10 early versus 4 late). Some 12 occurred after DBS system implantation and 2 after IPG replacement surgery (Table 2). No intracranial infections were found. The most common site for infection was a combination of the chest and connector site (6), followed by chest incision alone (3) as seen in Table 3. *Staphylococcus aureus *(MSSA) was the most common organism (Table 4). Intra-operative vancomycin powder was used in 187 implantation surgeries and 162 IPG replacement surgeries, with 10 (5.3%) infections after implantation and 1 (0.6%) infection after IPG replacement. Intra-operative vancomycin powder did not decrease infection risk after implantation (5.3% with versus 0.9% without) or IPG replacement (0.6% with versus 1.25% without) as seen in Table 2. Vancomycin powder use was shown to increase risk of infection after electrode implantation surgery (Relative Risk 5.5080, p = 0.02063). There was no statistical significant difference in choice of preoperative antibiotics due to significant difference in sample size with cefazolin being used in the majority of cases. Complete hardware removal occurred in eight patients, one patient had electrode only removal, three patients with I&D had no removal of hardware, and two patients with removal of IPG and extensor cables only. All patients were treated with intravenous antibiotics and no recurrent infections were found in patients with hardware left in place.

**Table 1:** Patient demographics, number of surgeries, and indications for surgery. IPG, Implantable Generator.

Demographics	
Number of patients	203
Mean age	71
Sex	128 males 75 females
Indication for surgery	Parkinson’s disease (152), essential tremor (47), dystonia (2), OCD(1), pain (1)
Total number of electrodes surgeries	391
Total number of IPG replacements	244
Total number of surgeries	635
Mean number of electrode surgeries	1.8
Mean number of IPG replacements	0.78

**Table 2:** Comparison of infections after implantation and implantable generator replacement, comparison of intra-operative vancomycin powder and preoperative antibiotics. IPG, Implantable Generator.

Comparison of Infections	
Total number of infections	14
Indication for surgery	Parkinson’s disease (152), essential tremor (47), dystonia (2), OCD (1), pain (1)
Early (less than 3 months) versus Late (greater than 3 months)	10 versus 4
Infections after implantation	12/391 (3%)
Infections after IPG replacement	2/244 (0.8%)
Infections after Implantation with intra-operative vancomycin powder versus without	10/187 (5.3%) versus 2/204 (0.9%) (p = 0.0138), Relative Risk= 5.0580 (p = 0.0263)
Infections after IPG replacement with intra-operative vancomycin powder versus without	1/162 (0.6%) versus 1/80 (1.25%) (p = 0.6105)
Infections after implantation with preoperative cefazolin versus vancomycin	10/368 (2.7%) versus 2/21 (9.5%) (p = 0.0841)
Infections after IPG replacement with preoperative cefazolin versus vancomycin	2/227 (0.88%) versus 0/15 (0%) (p = 0.7162)

**Table 3:** Infection sites.

Infection sites	Number of Infections
Cranial incision alone	2
Connector incision alone	2
Chest incision alone	3
Connector incision + chest Incision	6
Cranial incision + connector incision	1
Intracranial	0

**Table 4:** Infectious organisms isolated. Two patients had polymicrobial infections.

Organisms Isolated	Number of organisms isolated
Methicillin sensitive Staphylococcus aureus	5
Staphylococcus epidermidis	3
Methicillin resistant S. aureus	2
Pseudomonas aeruginosa	2
Proprioniobacterium acnes	1
Enterobacter aerogenes	1
Proteus mirabilis	1
Sterile culture	1

## Discussion

Our study demonstrates lower infection rates than reported historically in the literature (up to 25%) after both DBS implantation (3%) IPG replacement (0.8%) surgeries [2-5]. However, our study shows similar infection rates after implantation surgery compared to Abode-Lyamah et al. [2] which reported an infection rate of 3.7% and similar infection rates after IPG replacement compared to Pepper et al. [9] which had an infection rate of 0. In Abode-Lyamah et al. infection rates were higher during cases without use of intra-operative vancomycin powder use (9.7% versus 3.3%) but was not statistically significant after controlling for sex [2]. In our study, intra-operative use of vancomycin powder was not shown to decrease risk of infection after implantation or IPG replacement (Table 2). Vancomycin powder use was shown to increase risk of infection after electrode implantation surgery (Relative Risk 5.5080, p = 0.02063). This is in contrast to studies that reported a decrease in infections from 8.5% to 0% after MRSA screening and treating prior to IPG replacement as well as using intra-operative vancomycin powder during surgery [9]. Atchley et al. also showed that use of intra-operative vancomycin powder did not decrease infections during IPG surgery [10]. Our data support the current trends in the literature that shows that it is unclear that use of intra-operative vancomycin powder is beneficial.

Early infections were more common compared to late infections (10 versus 4). The earliest infection occurred one month postoperatively and the latest infection being six years postoperatively. The average number of electrode surgeries per patient was 1.8. Among the 12 patients with infections occurring after implantation surgery, the average number of prior electrode surgeries was 1.6. Seven patients sustained infections after the initial electrode implantation. Of the remaining five patients, the average number of prior electrode surgeries was 2.4. These results indicate that number of prior electrode implantation surgeries does not increase risk of infection. Among the two patients sustaining infections after IPG replacement, one had four prior IPG replacements while the other patient had two prior IPG replacements. Thus it is suggested that number of prior IPG replacement may be a risk factor for infection. Frequency and timing of IPG replacement depend on IPG type and stimulation settings which are related to nuclei being stimulated and condition being treated for each individual patient [11-12].

**Figure 1:**
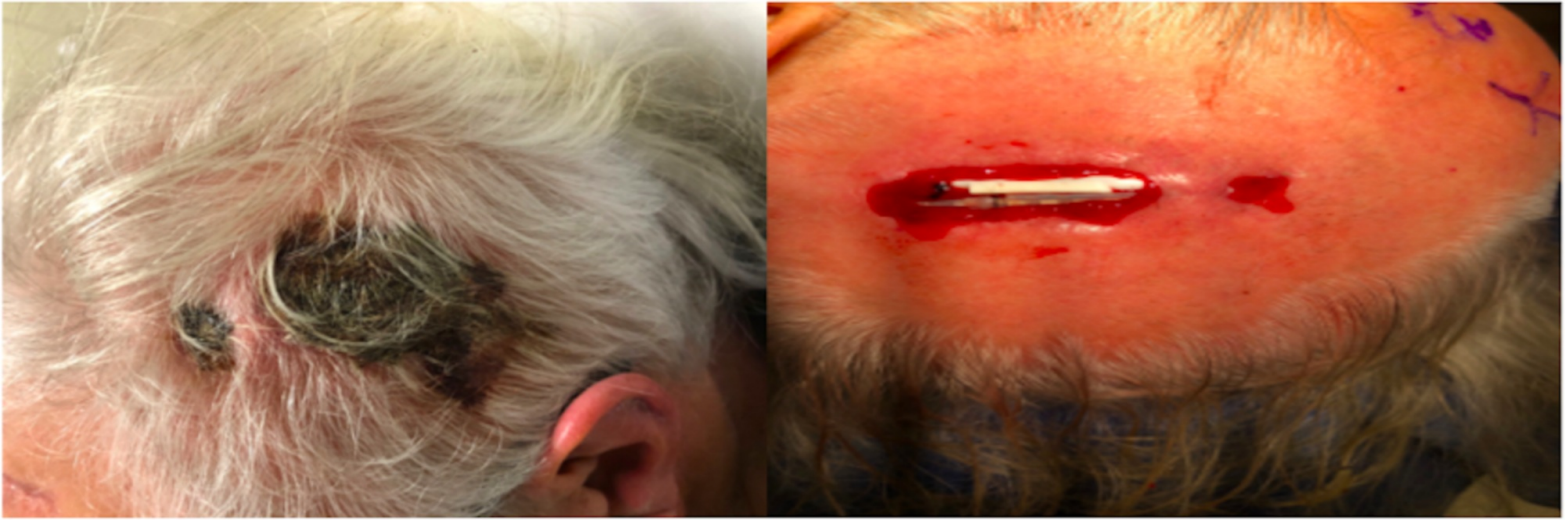
Infection at connector site-prior to surgical prep on left image and after surgical prep on right image.

The most common sites of infection were connector site and chest in combination in six patients, followed by the chest alone in three patients, and connector site alone in two patients (Table 3). The connector site is typically located in the posterior auricular region and often the scalp is very thin. The incision also tends to be a pressure point for the incision as patients often lay on this area while they rest. The connector sites vary by system with some creating more prominence than others. We postulate that the silicone boots covering the distal end of the electrode and the connector itself cause the skin erosion that leads to infection (Figure 1).

There were no intracranial infections present in this study. We believe this is due to early detection and treatment of any suspected infections. We also suspect that the use of bone cement to cover the burr hole may have acted as a barrier for infection to prevent tracking into the intracranial space. 

The most common organisms isolated were organisms routinely found in skin flora with *S. aureus* (MSSA) being most common, followed by S. epidermidis (Table 4). Antibiotic treatment and duration was tailored to the organisms isolated as well as the presence of residual DBS hardware. Complete removal of DBS hardware occurred in eight patients, one patient had removal of electrode only, two patients had removal of IPG and extensions only, and three patients had I&D without hardware removal. Two of the patients with no hardware removal were treated with long-term suppressive therapy. One patient who underwent complete removal had a subsequent re-implantation with no infection recurrence. Another patient who underwent complete removal with bilateral re-implantation developed another infection. However, this was attributed to a peripheral line infection rather than the procedure itself. The patient was admitted to the hospital for an unrelated problem and had positive blood cultures for MSSA due to an infected intravenous access site. No patients with hardware left in place developed a recurrence.

One limitation of our study is the lack of information regarding medical comorbidities that may have an increased infection risk such as diabetes, hypertension, drug use, smoking history, etc. However, 65 patients in our study were found to have diabetes. Of the 14 patients with infections, 6 patients had diabetes and intra-operative vancomycin was used in 4 patients. However, information regarding assessment of glucose control in the patients such as HbA1c at time of surgery or time of infection was unavailable. Further investigation into medical risk factors such as diabetes for DBS infections is warranted.

Another limitation of our study is that many choices are based on surgeon preference such as which peri-operative antibiotic was used and whether DBS hardware was removed or not. A prospective study would provide a better comparison of infection rates after use of intra-operative vancomycin powder. 

## Conclusions

The most common causative organism in our DBS infections was MSSA. Infections after DBS implantation and IPG replacement occurred in 3% and 0.8% of patients in our study respectively, which is lower than reported historically. Intra-operative use of vancomycin was not shown to decrease risk of infection after electrode implantation surgery or IPG replacement. However, in our study it was shown to increase risk of infection after electrode implantation surgery. Treatment includes tailored antibiotic therapy and surgical debridement with or without hardware removal. DBS hardware can be safely left in place in select patients on a case by case basis who may have significant adverse effects if it is removed. 
